# Mechanism of Icariin induces mitochondrial-mediated apoptosis by Wnt/β-catenin signaling pathway in colorectal cancer

**DOI:** 10.3389/fphar.2026.1857797

**Published:** 2026-08-06

**Authors:** Xin Wang, Rui-Yun Lu, Hao-Bo Wang, Yi-Jiong Li, Li-Fang Guo, Tian-Hao Lan, Chao Pang, Cong-Hui Wang, Jing Zhang, Fang-Jian Shang, Li-Min Meng, Zeng-Ren Zhao

**Affiliations:** 1 Department of General Surgery, Hebei Key Laboratory of Colorectal Cancer, The First Hospital of Hebei Medical University, Shijiazhuang, China; 2 Department of Pathology, The First Hospital of Hebei Medical University, Shijiazhuang, China; 3 Department of Traditional Chinese Medicine, The First Hospital of Hebei Medical University, Shijiazhuang, China; 4 Department of Breast and Thyroid Center, The First Hospital of Hebei Medical University, Shijiazhuang, China; 5 Department of Health Management Center, The First Hospital of Hebei Medical University, Shijiazhuang, China; 6 Department of Spleen-Stomach Diseases, Hebei Province Hospital of Chinese Medicine, Shijiazhuang, China; 7 Department of Gastrointestinal Disease Diagnosis and Treatment Center, The First Hospital of Hebei Medical University, Shijiazhuang, China; 8 Department of Nursing, The First Hospital of Hebei Medical University, Shijiazhuang, China

**Keywords:** colorectal cancer, Icariin, mitochondrial-mediated apoptosis, molecular mechanism, Wnt/β-catenin signaling pathway

## Abstract

**Background:**

To evaluate the effect of Icariin (ICA) on AOM/DSS-induced colorectal cancer (CRC) in mice as well as CRC cells, and to explore the underlying molecular mechanism.

**Methods:**

The therapeutic efficacy of ICA against AOM/DSS-induced CRC was evaluated by detecting tumor growth and pathological alterations. Biomarkers related to Wnt/β-catenin and mitochondrial apoptosis were tested, and cell experiments were performed to validate the regulatory correlation between ICA and the Wnt/β-catenin pathway in CRC cells.

**Results:**

ICA significantly reduced tumor number, size and ameliorated pathological damage in AOM/DSS-induced mice by regulating the mitochondrial-mediated apoptosis of colon tissue. ICA’s regulation of mitochondrial-mediated apoptosis was confirmed to involve the Wnt/β-catenin signaling pathway. *In vitro*, ICA restrained proliferation, migration and invasion of SW1463 and HCT116 cells, decreased mitochondrial membrane potential and changed the expression of pathway-related genes.

**Conclusion:**

The study demonstrated that ICA promoted mitochondrial-mediated apoptosis by regulating the Wnt/β-catenin signaling pathway, suggesting that ICA could serve as a potential treatment for CRC.

## Introduction

Colorectal cancer (CRC) is a prevalent malignancy characterized by the abnormal proliferation of cells persistently within the intestinal cavity. It ranks third globally in terms of incidence among malignant tumors and second in mortality (Sung et al., 2021). The rising incidence of CRC can be largely attributed to changes in dietary habits and lifestyle, thereby imposing a significant societal burden (Surti et al., 2022; Siegel et al., 2020). Notably, CRC typically has an insidious onset, with pronounced symptoms often manifesting only at advanced stages. Consequently, many patients are diagnosed at a point where surgical intervention is no longer viable, leaving chemotherapy and adjuvant treatments such as immunotherapy and biological agents as the primary therapeutic options, albeit with limited efficacy (Simard et al., 2019; Biller and Schrag, 2021; Yang W. et al., 2023). Chemotherapy resistance frequently arises in a subset of patients due to tumor heterogeneity and individual variability (Kuipers et al., 2015). Consequently, there is an urgent need to develop novel and more efficacious agents for the prevention and treatment of CRC.

Apoptosis is a conserved programmed cell death that plays an indispensable role in balancing cell growth and tissue homeostasis, which eliminates aged, impaired and redundant cells to guarantee physiological tissue renovation. Mitochondria dominate the intrinsic apoptotic cascade, and mitochondrial-originated apoptosis occupies a core position among diverse apoptotic patterns (Kłos and Dabravolski, 2021). Diverse adverse stimuli such as oxidative stress, infection and irradiation can destroy mitochondrial structural integrity and trigger the discharge of pro-apoptotic factors to initiate downstream apoptotic events (Zhou et al., 2023). Since escaping apoptotic elimination is a typical malignant feature enabling sustained proliferation of colorectal cancer cells after DNA injury (Shi et al., 2023), pharmacological induction of tumor apoptosis has become a feasible therapeutic direction for CRC intervention.

Icariin (ICA), a flavonoid, is a natural compound extracted from the stems and leaves of Epimedium brevicornu Maxim. An increasing number of studies have shown that ICA has significant anti-tumor effect (Zhang C. et al., 2023). Recently, a study suggested that ICA induced cell apoptosis in breast cancer by inhibiting the JNK/c-Jun pathway (Shi et al., 2022). In addition, other research has showed that ICA could inhibit the tumorigenesis in ovarian cancer by activating mitochondria-dependent pathway (Lei et al., 2025). However, few studies have elucidated that ICA promotes cell apoptosis from the perspective of mitochondrial intrinsic pathway in colon cancer. Therefore, in the present study, we evaluated the therapeutic effects of ICA on CRC by using an AOM/DSS-induced CRC mouse model and explored how ICA could induce cell apoptosis. In addition, we further cultured HCT116 and SW1463 cells to observe the ability of ICA to anti-tumor and to verify the relevant mechanisms.

## Materials and methods

### Animals

SPF male C57BL/6J mice (6–8 weeks age, 18–22 g) were purchased from Beijing SiPeiFu Biotechnology Co., Ltd. [Licence No: SYXK (Jing)2019–0010]. The C57BL/6J mice were maintained under SPF conditions with temperature (22 °C ± 2 °C), humidity (55% ± 5%), and controlled photoperiod (12-h light/dark cycles). This animal experiment was performed strictly in compliance with disciplines of the National Institutes of Health guide for the care and use of laboratory animals, and approved by the Experimental Animal Ethics Committee (No. DWLL202401013).

### Establishment of the CRC model and treatment

The CRC mice model was prepared by the Azoxymethane (AOM, Sigma-Aldrich, California, United States)/Dextran sulfate sodium (DSS, MP Biomedicals, California, United States). A large number of animal experiments have confirmed that this method is effective for modeling (Wu et al., 2024; Gou et al., 2023). After 7 days of acclimation, the 60 C57BL/6J mice were randomly divided into six groups (n = 10 for each group): normal group, model group, ICA -L group (25 mg/kg), ICA -M group (50 mg/kg), ICA -H group (100 mg/kg), and ASA group (15 mg/kg). The normal group was given regular distilled water for the entire experiment. In the other experimental groups, AOM (10 mg/kg) was intraperitoneally injected once, and after AOM administration for 1 week, 2% DSS solution was given for consecutive 1 week, followed by normal distilled drinking water for 2 weeks. Drinking DSS solution alternated with normal distilled drinking water for a total of 3 cycles.

ICA and ASA were given intragastric administration to the respective groups from the day of AOM intervention. Following the 11 weeks gavage, the mice were deprived of water for 24 h. Dissection was carried out, and a cecum-anal sample was removed. This removal was followed by opening the mouse longitudinally and photographic documentation, as well as measurements of tumor numbers and size. The intestinal tumor tissue samples were rapidly cut and fixed in 4% glutaraldehyde for electron microscope observation. One part was preserved in 4% paraformaldehyde, and the other part was transferred to liquid nitrogen, and subsequently transferred to a refrigerator stored at −80 °C.

### Hematoxylin-eosin (HE) staining and immunohistochemistry (IHC)

Part of the colorectal tissue was fixed with 4% paraformaldehyde solution and sectioned in paraffin at 5 μm thickness for HE staining. Sections were deparaffinized with xylene and high-temperature antigen retrieval with sodium citrate buffer solution. IHC was performed using antibodies. After, the color reaction of sections was visualized using DABs (Servicebio, Wuhan, China) chromogenic kits.

### Quantitative real-time polymerase chain reaction (qRT-PCR)

Total RNA was extracted by TRLzol Reagent (Aidlab Biotechnologies, Beijing, China) and synthesized to cDNA using a reverse transcription kit (GeneCopoeia, Guangzhou, China). qRT-PCR amplification was performed using a 2×SYBR Green qPCR Master Mix kit (Servicebio, Wuhan, China). The relative expression of genes was analyzed using the 2^−ΔΔCt^ quantitate method. The primers are shown in Table 1.

**TABLE 1:** Primer sequence.

Gene name	Primer sequence	Product length/bp
Bcl-2	F: TGACTTCTCTCGTCGCTACCGT R: CCTGAAGAGTTCCTCCACCACC	120
Bax	F: TGG​TTG​CCC​TCT​TCT​ACT​TTG​C	100
	R: AAG​TCC​AGT​GTC​CAG​CCC​AT	
Cyto C	F: CCA​ACA​AGA​ACA​AAG​GCA​TCA​C	126
	R: CTG​CCC​TTT​CTC​CCT​TCT​TCT​TA	
Caspase-3	F: TAA​AGA​CCA​TAC​ATG​GGA​GCA​AG	156
	R: CCA​CAT​CCG​TAC​CAG​AGC​GA	
Wnt	F: TGA​CTC​GCA​TCA​TAA​GGG​GC	181
	R: GTG​GTC​CAG​GAT​AGT​CGT​GC	
β-catenin	F: GCT​GCT​GTC​CTA​TTC​CGA​ATG​TCT​G	141
	R: GGC​ACC​AAT​GTC​CAG​TCC​AAG​ATC	
CyclinD1	F: GAG​GCG​GAG​GAG​AAC​AAA​CA	96
	R: GGA​GGG​CGG​ATT​GGA​AAT​GA	
C-myc	F: CCTAGTGCTGCATGAGGAGACAC R: TCCACAGACACCACATCAATTTCTT	93
GAPDH	F: CCT​CGT​CCC​GTA​GAC​AAA​ATG	133
	R: TGA​GGT​CAA​TGA​AGG​GGT​CGT	

### Western blotting (WB)

According to the instructions of the manufacturer, the total protein of cells was extracted with RIPA lysate (Servicebio, Wuhan, China). Afterward, SDS-PAGE (Boster, Wuhan, China) was prepared according to the equivalent protein concentrations. Next, the proteins were electrophoretically transferred from the gel to PVDF membranes (Servicebio, Wuhan, China). After the membranes were blocked with 5% nonfat milk, and incubated with the primary antibodies at 4 °C overnight. Finally, the membranes were incubated with secondary antibodies at room temperature for 2h, and protein bands were detected by a gel imaging analysis system.

### TUNEL assay

Apoptosis was evaluated by TUNEL assay according to the manufacturer’s instructions. The sections were placed in a wet box and incubated with TUNEL reaction solution at 37 °C for 1 h. After washing 3 times with PBS, the sections were incubated with DAPI dye solution at room temperature for 10 min away from light. The apoptotic cells were observed with green fluorescence and the images were collected by fluorescence microscope (Nikon, Tokyo, Japan, Nikon Eclipse).

### Transmission electron microscopy

The colorectal tumor tissues were harvested and fixed in an electron microscope fixative solution, then they were fixed in 1% osmium and phosphate buffer for 2 h. Tumor tissues were dehydrated, embedded, sectioned, and stained, then tissues were observed by a transmission electron microscope (HITACHI, Tokyo, Japan, HT7800).

### RNA sequencing

RNA sequencing of the colon tissue was performed by Personalbio (Nanjing, China). Total RNA was extracted using Trizol reagent and detected evaluating contamination, purity, and integrity. A transcriptome library was constructed according to the manufacturer’s instructions and evaluated based on the mean mass distribution of reads. After the raw data was recorded in FASTQ format and gene expression levels were normalized and presented as FPKM. The screening criteria for differentially expressed genes (DEGs) were fold change (FC) > 2 with p-value<0.05. Functional enrichment analysis was performed employing the Kyoto Encyclopedia of Genes and Genomes (KEGG) database.

### Cell culture

The human CRC cell lines SW1463 and HCT116 were obtained from the First Hospital of Hebei Medical University (Hebei, China). SW1463 cells were routinely cultured in DMEM medium (Gibco, Oklahoma, United States) supplemented with 10% fetal bovine serum (Gibco, Oklahoma, United States) and 1% penicillin/strepto-mycin (Gibco, Oklahoma, United States) and HCT116 cells were routinely cultured in McCoy’s 5A medium (Gibco, Oklahoma, United States) supplemented with 10% fetal bovine serum and 1% penicillin/strepto-mycin. The SW1463 and HCT116 cells were seeded in a 96-well plate for cell treatment and pretreated with ICA for 2 h at 37 °C with 5% CO_2_. After 2 h, SW1463 and HCT116 cells were treated with ICA for 48 h.

### Cell viability assay

Cell viability was detected using Cell Counting Kit-8 (CCK-8, Boster, Wuhan, China). Logarithmic SW1463 and HCT116 cells were seeded into 96-well plates at 1 × 10^3^/well and were treated with different concentrations of ICA. After 72 h each well was added to 10 µL CCK-8 solution, and incubated for 2 h at 37 °C. The absorbance at a wavelength of 450 nm was detected by a microplate reader.

### Colony formation assays

Cells were seeded in a 6-well plate at 1 × 10^3^/well and incubated for 24 h. After cells were treated with different concentrations of ICA for 14 days. Subsequently, the cells were fixed with 4% paraformaldehyde for 15 min, and stained with 0.5% crystal violet for another 15 min. Then fixed-sized colonies were counted under an optical microscope.

### Transwell migration and invasion assay

DMEM supplemented with 10% FBS in different groups was placed into the lower chamber. Serum-free medium with tumor cells was added to the upper chamber. The cells were treated for 24 h at 37 °C. the lower chamber was loaded with 4% paraformaldehyde and 0.1% crystal violet solution. The chamber was counted in five random fields. The transwell migration experiment was performed according to the above steps, but matrigel was used for the upper chambers inserted in the transwell invasion experiment.

### Flow cytometry assay

The cell apoptosis was measured using an Annexin V-FITC Apoptosis Detection Kit (Seven, Beijing, China). 1 × 10^6^/well cells were seeded into a 6-well plate and incubated overnight. After the cells were washed and resuspended with binding buffer and then stained with Annexin V/propidium iodide (PI) at room temperature for 10 min. The levels of apoptosis were detected using a BD FACSCalibur™ Flow Cytometer (BD Bio-sciences, United States, no. E97501093).

### Mitochondrial membrane potential detection assays

Mitochondrial membrane potential was assessed using a mitochondrial membrane potential assay kit with a lipophilic fluorochrome known as JC-1 (Servicebio, Wuhan, China). Logarithmic cells were seeded into a 24-well plate and incubated for 24 h. Then each well was added to 0.5 mL JC-1staining solution, and incubated for 20 min at 37 °C. After incubation, the cells were washed with PBS buffer for twice, and photographed by fluorescence microscope.

### Statistical analysis

All statistical analyses were performed using SPSS 21.0 (SPSS Inc., Chicago, United States). All data were described with the mean ± standard deviation (SD). One-way ANOVA was used for multiple comparisons, and *P* < 0.05 represented statistical significance. GraphPad Prism 9.0 (GraphPad Software, San Diego, United States) was used to construct a graph.

## Results

### ICA suppresses CRC in AOM/DSS-induced mice

**FIGURE 1:**
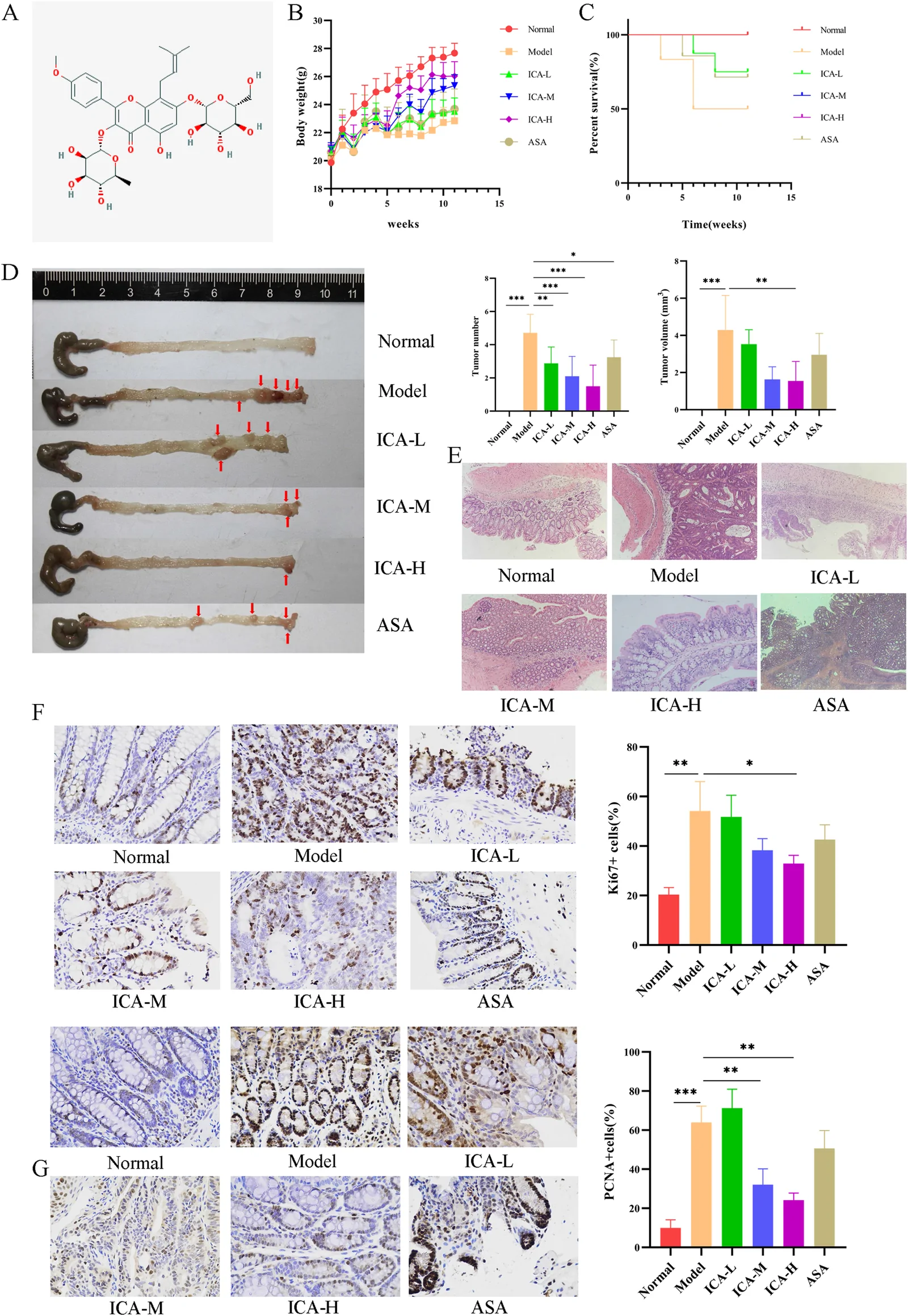
ICA suppresses CRC in AOM/DSS-induced mice. **(A)** Chemical structure of ICA. **(B)** Body weight change. **(C)** Survival rate. **(D)** Tumor number and size. **(E)** HE staining. **(F)** Immunochemistry staining for Ki67. **(G)** Immunochemistry staining for PCNA.

To explore the therapeutic effect of ICA on colorectal tumorigenesis, we gavaged AOM/DSS-induced C57BL/6 mice with ICA. The molecular structure of ICA is shown in Figure 1A. CRC mice exhibited major body weight loss compared to the normal group, while the body weight was significantly increase in ICA (Figure 1B). In addition, compared with the model group, the ICA group had significantly increased survival rate (Figure 1C). Based on the statistical results, the number and size of tumor in model group were increased, whereas the normal group showed fewer tumors and smaller tumor size (Figure 1D). The histopathological results showed that the colonic crypt structure is disturbed, the original tubular structure is lost, some glands are atrophied and necrotic, the nucleus is atypical, and the lumen surface shows high-grade intraepithelial tumor-like changes in the model group. After ICA treatment, the colon histopathological characteristics were improved (Figure 1E). Additionally, the number of Ki67-positive cells and PCNA-positive cells were significantly increased in CRC mice compared with the normal group, while the cell proliferation was reduced by treatment with ICA (Figures 1F,G).

### ICA promotes the mitochondrial-mediated apoptosis in CRC

**FIGURE 2:**
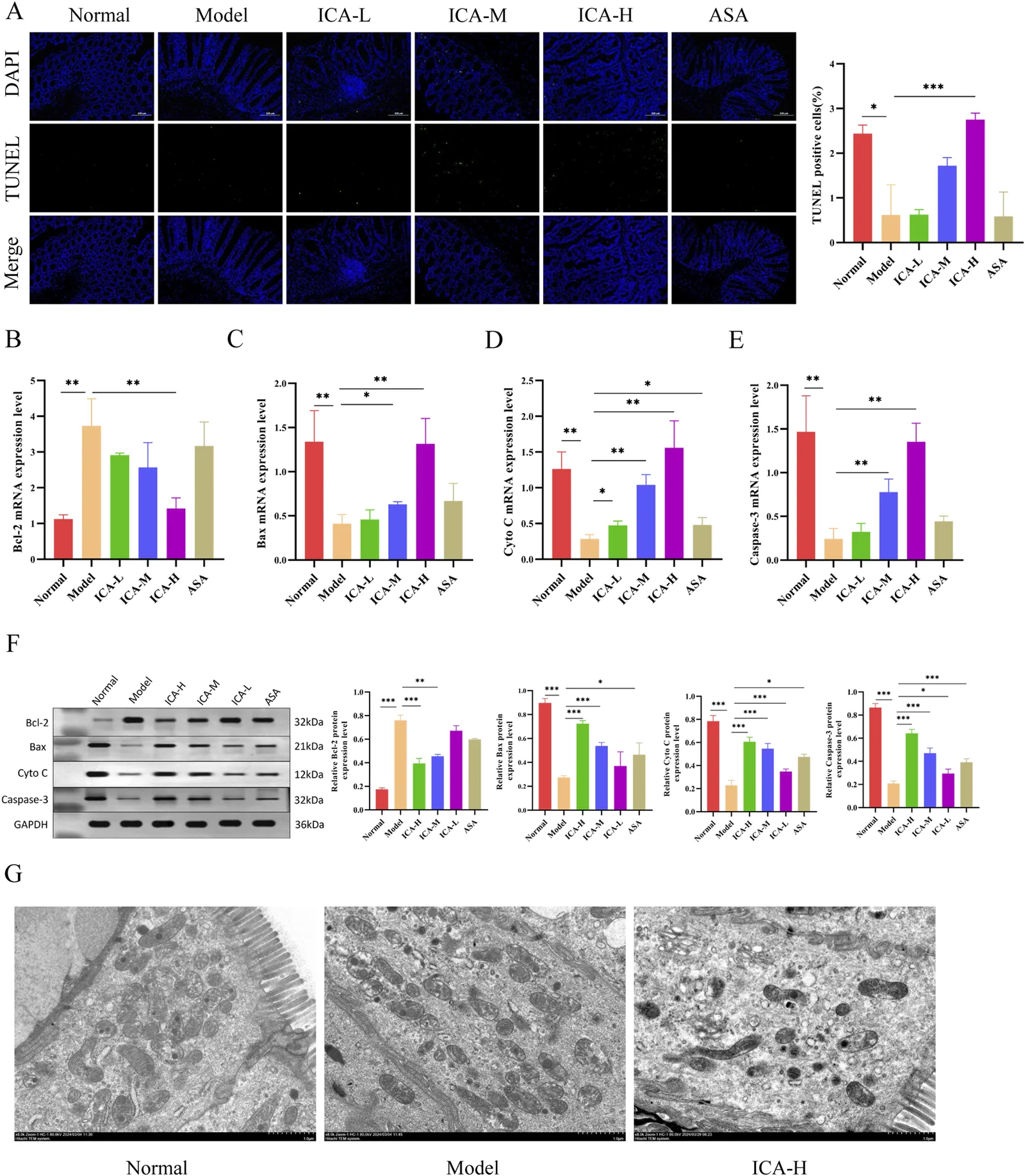
ICA promotes the mitochondrial-mediated apoptosis in CRC. **(A)** Immunofluorescence staining for TUNEL. **(B)** mRNA level of Bcl-2. **(C)** mRNA level of Bax. **(D)** mRNA level of Cyto C. **(E)** mRNA level of Caspase-3. **(F)** Protein levels of Bcl-2, Bax, Cyto C, and Caspase-3. **(G)** Transmission electron microscopy of mitochondrial morphology.

The mitochondrial pathway is the core of the intrinsic apoptosis pathway, promoting mitochondrial-mediated apoptosis plays a key role in inhibiting the excessive proliferation of tumor cells. First, we evaluated tumor cell apoptosis using the TUNEL assay. The results indicated that the number of TUNEL-positive cells was reduced in CRC mice compared with the normal group, but it was significantly upregulated by ICA treatment (Figure 2A). In addition, we evaluated the expression levels of mitochondrial apoptosis-related genes and proteins in different groups, including Bcl-2, Bax, Cyto C, and Caspase-3. The results indicated that the mRNA expression levels of Bax, Cyto C, and Caspase-3 decreased and Bcl-2 increased in CRC mice compared with the normal group, but Bax, Cyto C, and Caspase-3 significantly increased and Bcl-2 decreased by ICA treatment (Figures 2B–F). Furthermore, we observe the effect of ICA on mitochondrial morphology by electron microscopy. The results revealed more mitochondria in the model group, and their size increased with a slightly expanded ridge. After ICA -H treatment, the mitochondria ridges were not clear, and they were slightly dissolved showing vacuolar changes (Figure 2G).

### ICA promotes mitochondrial-mediated apoptosis by inhibiting Wnt/β-catenin signaling pathway

**FIGURE 3:**
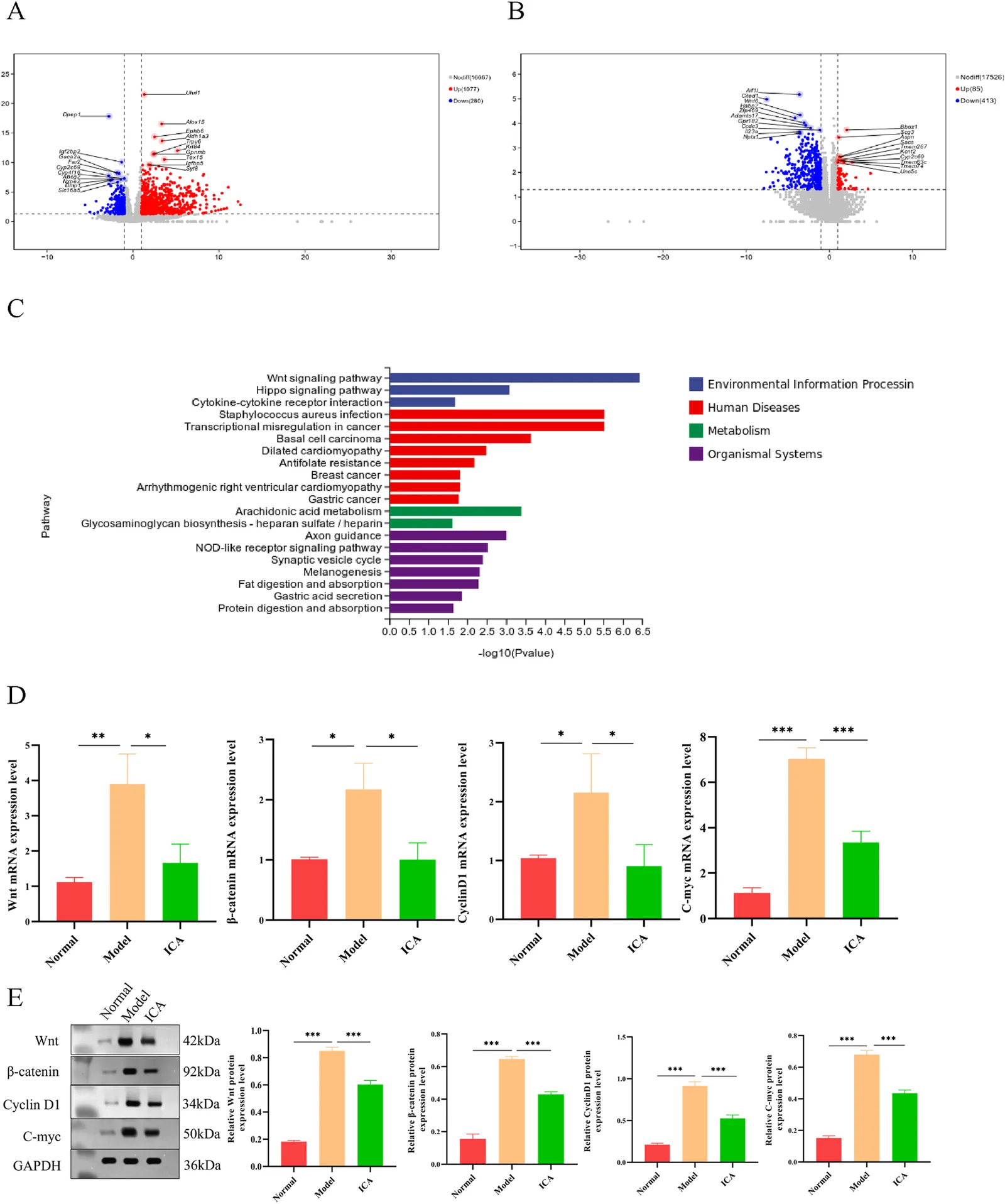
ICA promotes mitochondrial-mediated apoptosis by inhibiting Wnt/β-catenin signaling pathway. **(A)** Volcano plot analysis of DEGs in the model vs. normal groups. **(B)** Volcano plot analysis of DEGs in the ICA-H vs. model groups. **(C)** KEGG analysis of interacting DEGs in the three groups. **(D)** qRT-PCR verification of DEGs related to the Wnt/β-catenin signaling pathway. **(E)** WB verification of DEGs related to the Wnt/β-catenin signaling pathway.

To further investigate the underlying mechanism by which ICA regulates mitochondrial-mediated apoptosis under CRC conditions, RNA sequencing was performed on colorectal cancer tissue. DEGs were screened according to the thresholds of FC > 2 and p-value<0.05. Overall, compared with the normal group, a total of 1,077 upregulated and 280 downregulated DEGs were identified in the model group. Compared with the model group, a total of 85 upregulated and 413 downregulated DEGs were identified in the ICA group (Figures 3A,B). Moreover, a total of 46 intersecting targets were obtained. KEGG enrichment analysis showed that the interacting DEGs in the three groups were mainly enriched in Wnt signaling (Figure 3C). According to the RNA-seq results, we speculated that ICA regulated mitochondrial-mediated apoptosis by Wnt signaling. Furthermore, qRT-PCR validated the relative expression of DEGs enriched in Wnt signaling, such as Wnt, β-catenin, CyclinD1, and C-myc, and the results were consistent with the RNA-seq results. The ICA group showed decreased gene levels of Wnt, β-catenin, CyclinD1 and C-myc compared to the model group (Figure 3D). Meanwhile, the ICA group showed decreased protein levels of Wnt, β-catenin, C-myc and CyclinD1compared to the model group (Figure 3E).

### ICA repressed proliferation, migration, invasion, mitochondrial membrane potential levels and promoted apoptosis of CRC cells

**FIGURE 4:**
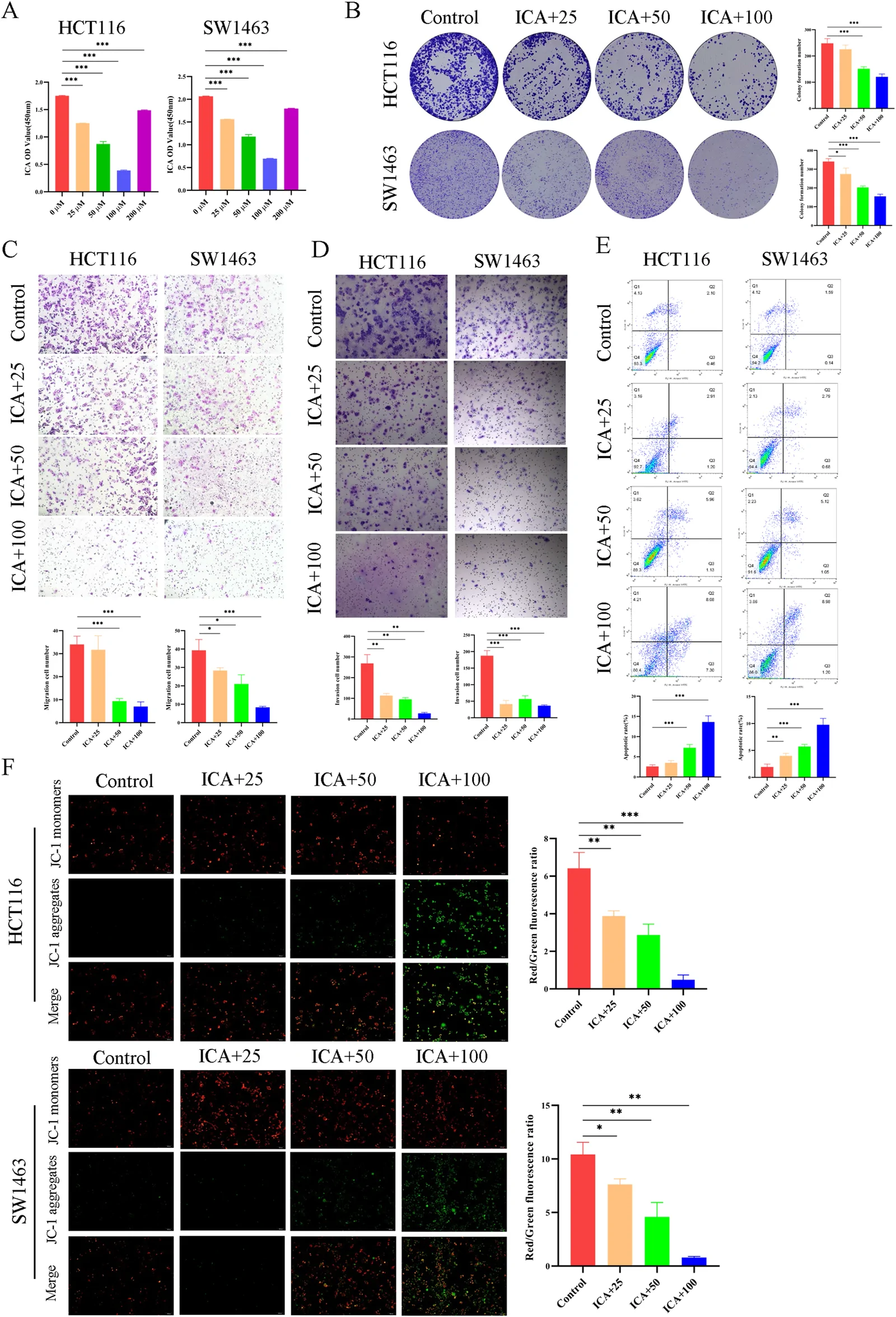
ICA repressed proliferation, migration, invasion, mitochondrial membrane potential levels and promoted apoptosis of CRC cells. **(A)** CCK-8 results. **(B)** Colony formation results. **(C)** Invasion results. **(D)** Migration results. **(E)** Flow cytometry results. **(F)** JC-1 staining for mitochondrial membrane potential.

To investigate the effects of ICA on CRC cells, we chose the ICA doses (25 μM, 50 μM, and 100 μM) according to the CCK-8 results (Figure 4A). Colony formation assays, transwell migration and invasion assay were performed to assess cell proliferation, migration, and invasion of CRC cell lines. As shown in Figures 4B–D, ICA significantly inhibited the proliferation, migration, and invasion of SW1463 and HCT116 cells. In addition, flow cytometry showed that ICA promoted apoptosis (Figure 4E). Additionally, mitochondrial membrane potential showed that ICA significantly reduced mitochondrial membrane potential levels (Figure 4F).

### ICA promotes the mitochondrial-mediated apoptosis of CRC cells by inhibiting Wnt/β-catenin signaling pathway

**FIGURE 5:**
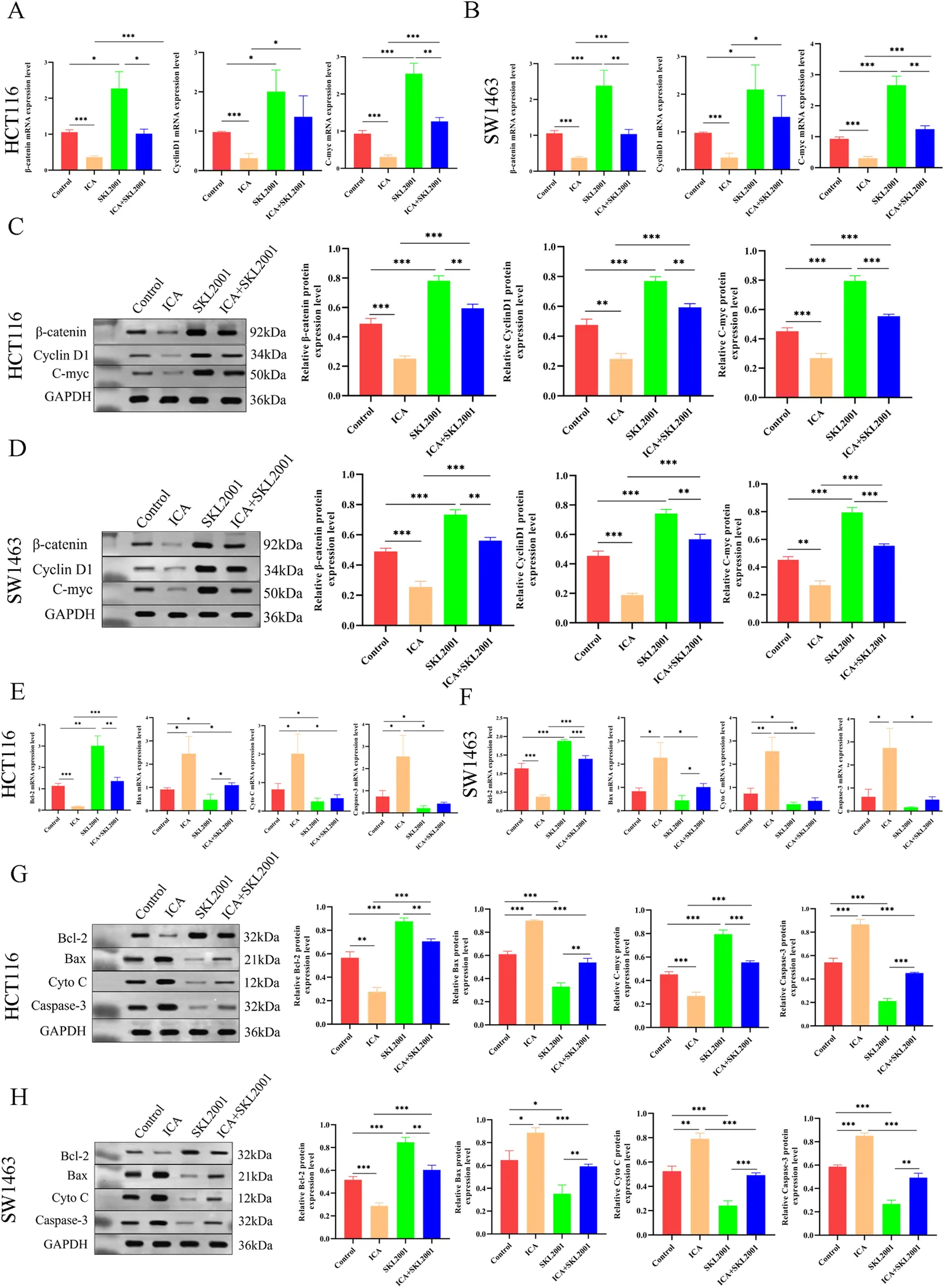
ICA promotes the mitochondrial-mediated apoptosis of CRC cells by inhibiting Wnt/β-catenin signaling pathway. **(A)** mRNA level of β-catenin, CyclinD1, and C-myc in HCT116. **(B)** mRNA level of β-catenin, CyclinD1, and C-myc in SW1463. **(C)** Protein levels of β-catenin, CyclinD1, and C-myc in HCT116. **(D)** Protein levels of Bax, Cyto C, and Caspase-3 in SW1463. **(E)** mRNA level of Bcl-2, Bax, Cyto C, and Caspase-3 in HCT116. **(F)** mRNA level of Bcl-2, Bax, Cyto C, and Caspase-3 in SW1463. **(G)** Protein levels of Bcl-2, Bax, Cyto C, and Caspase-3 in HCT116. **(H)** Protein levels of Bcl-2, Bax, Cyto C, and Caspase-3 in SW1463.

To further prove whether ICA regulates mitochondrial-mediated apoptosis through Wnt signaling, we added the Wnt signaling agonist SKL2001 to SW1463 and HCT116 cells and measured the expression levels of β-catenin, CyclinD1, C-myc, Bcl-2, Bax, Cyto C, and Caspase-3 by qRT-PCR and WB. As shown in Figures 5A–D, the ICA could effectively suppress mRNA and protein expression levels of β-catenin, CyclinD1, and C-myc and SKL2001 significantly increased the mRNA and protein expression levels of β-catenin, CyclinD1 and C-myc. In addition, the inhibitory effect of ICA on Wnt signaling was attenuated by SKL 2001. Moreover, ICA increased mRNA levels of Bax, Cyto C, and Caspase-3 and decreased Bcl-2, SKL2001 significantly suppressed the mRNA level of Bax, Cyto C, and Caspase-3 and decreased Bcl-2, and the expression level of ICA on mitochondria-related pathways was reversed by SKL2001 (Figures 5E,F). Meanwhile, ICA increased the protein levels of Bax, Cyto C, and Caspase-3 and decreased Bcl-2, SKL2001 significantly suppressed the protien levels of Bax, Cyto C, and Caspase-3 and increased Bcl-2, and the expression level of ICA on mitochondria-related pathways was reversed by SKL 2001 (Figures 5G,H).

## Discussion

CRC ranks among the most common malignant tumors of the digestive tract worldwide and is a leading contributor to cancer-related mortality, seriously endangering public health (Ruan et al., 2020). Conventional clinical regimens including surgical resection, radiotherapy and chemotherapy still face bottlenecks such as limited overall therapeutic efficiency. Therefore, it is imperative to develop innovative candidate agents for the prevention and clinical intervention of CRC. In the current research, we explored the anti-CRC activity of ICA together with its underlying molecular mechanism. *In vivo* results demonstrated that ICA treatment effectively diminished tumor burden and alleviated colon pathological lesions in AOM/DSS-induced CRC model mice. Mechanistically, ICA triggers mitochondrial-dependent apoptotic cascades via suppressing aberrant Wnt/β-catenin signaling activation, indicating its promising potential as a candidate anti-CRC agent. Subsequent cellular validation based on SW1463 and HCT116 cell lines further corroborated the above pharmacological effect and related molecular mechanism.

Apoptosis is a precisely orchestrated programmed cell death essential for sustaining physiological tissue homeostasis in multicellular organisms (Cheng and Ferrell, 2018). Dysregulated apoptotic signaling represents a core hallmark of malignant transformation, enabling tumor cells to escape apoptotic clearance and sustain uncontrolled proliferation across multiple malignancies including CRC (Mohammad et al., 2015). Accordingly, pharmacological induction of tumor cell apoptosis has become a vital therapeutic strategy for CRC intervention (Vaghari‐Tabari et al., 2021). Three canonical apoptotic cascades have been characterized: the mitochondrial intrinsic pathway, the death receptor-initiated extrinsic pathway, and the endoplasmic reticulum stress-dependent apoptotic pathway. As indispensable organelles governing energy metabolism and cell fate, mitochondria serve as the central regulatory hub of the intrinsic apoptotic cascade (Yin and Zhu, 2012). Upon structural damage, cytoplasmic Bax translocates to the outer mitochondrial membrane and facilitates pore formation, which prompts cytochrome C release into the cytosol and sequentially activates downstream caspase cascades to execute mitochondrial-originated apoptosis (Badrinath and Yoo, 2018; Czabotar et al., 2013; Bock and Tait, 2019). Consistent with these theoretical foundations, our *in vivo* data validated the pro-apoptotic property of ICA. ICA administration markedly elevated TUNEL-positive apoptotic cell ratio in colonic tumor tissues, upregulated pro-apoptotic biomarkers (Bax, cytochrome C, Caspase-3) while suppressing anti-apoptotic Bcl-2 expression. Ultrastructural observation further revealed mitochondrial morphological abnormalities in ICA-treated groups, including blurred mitochondrial cristae, partial organelle lysis and cytoplasmic vacuolation. Collectively, phenotypic, molecular and ultrastructural evidence consistently demonstrates that ICA triggers tumor cell death via activation of the mitochondrial apoptotic program.

Mitochondria-dependent intrinsic apoptosis is modulated by multiple upstream stimuli such as oxidative stress, collapse of mitochondrial membrane potential, protein mitochondrial translocation and cell cycle remodeling, which are governed by coordinated signaling networks (Badrinath and Yoo, 2018; Hsu et al., 2014). Uncovering how ICA restores apoptotic sensitivity in apoptosis-resistant CRC cells helps to advance new therapeutic strategies for CRC management. In the present study, whole-transcriptome profiling via RNA-seq was performed to screen differentially expressed genes in ICA-treated CRC mouse colon tissues. Subsequent KEGG pathway enrichment prominently implicated aberrant Wnt/β-catenin cascade as a core candidate pathway underlying ICA-initiated mitochondrial apoptosis. Accumulating literature have validated that Wnt/β-catenin signaling exerts critical regulatory functions on mitochondrial homeostasis and intrinsic apoptotic cascades (Chen et al., 2022). Pharmacological suppression of Wnt/β-catenin compromises mitochondrial structural integrity, accelerates efflux of pro-apoptotic mediators and eventually triggers mitochondrial-dependent tumor cell apoptosis (Li et al., 2015). Conversely, excessive Wnt/β-catenin activation transcriptionally upregulates its downstream targets including CyclinD1 and c-Myc; elevated CyclinD1 drives G1/S cell-cycle transition and accelerates malignant proliferation, accompanied by mitochondrial membrane potential elevation and suppressed mitochondrial apoptosis. Such dysregulation breaks the physiological balance between proliferation and programmed cell death and fuels uncontrolled hyperproliferation of colonic epithelial cells (Song et al., 2024; Jamasbi et al., 2022). Consistent with above reports, our *in vivo* data demonstrated that ICA markedly downregulated the protein expression of Wnt, β-catenin, c-Myc and CyclinD1 in colon tumor tissues from AOM/DSS-induced CRC mice. Taken together, we reasonably conclude that ICA facilitates mitochondrial apoptosis in CRC predominantly via suppressing abnormal Wnt/β-catenin signaling.

Uncontrolled proliferation, apoptotic resistance, and enhanced migratory and invasive capacities are the core malignant hallmarks of tumor cells. To further validate the anti-tumor efficacy of ICA at the cellular level, two typical CRC cell lines (HCT116 and SW1463) were adopted for *in vitro* functional verification. Cellular experimental results demonstrated that ICA treatment significantly suppressed the proliferation, migration and invasion of CRC cells, reduced mitochondrial membrane potential, and ultimately elevated the apoptotic rate of tumor cells. Collectively, these *in vitro* findings further corroborate the potent anti-tumor activity of ICA against CRC malignant phenotypes.

Accumulating evidence has confirmed that core components of the Wnt signaling pathway serve as critical regulators of mitochondrial mass and mitochondrial membrane potential (Yang Y. et al., 2023). Our *in vivo* experiments in CRC model mice validated that ICA exerts pro-mitochondrial apoptosis effects via modulating the Wnt/β-catenin signaling cascade. Consistently, a prior study has reported that ethylbenzene induces mitochondrial-dependent apoptosis by suppressing Wnt/β-catenin activation in CRC, which supports our mechanistic conclusion (Zhang M. et al., 2023). To further solidify the regulatory mechanism of ICA on mitochondrial apoptosis at the cellular level, we detected the expression levels of Wnt, β-catenin, CyclinD1, and c-Myc in CRC cells. The *in vitro* molecular alterations were highly consistent with our *in vivo* experimental results. Notably, pharmacological activation of Wnt/β-catenin signaling by the specific agonist SKL2001 markedly reversed the inhibitory effect of ICA on β-catenin, CyclinD1, and c-Myc expression. Furthermore, SKL2001-mediated Wnt pathway activation significantly abrogated ICA-induced mitochondrial apoptosis in CRC cells. Collectively, these rescue assay results definitively verify that ICA promotes mitochondrial-dependent apoptosis and suppresses CRC malignant progression through targeted inhibition of the Wnt/β-catenin signaling pathway.

This study has several limitations. First, although sufficient experimental data demonstrate that ICA exerts anti-CRC effects via regulating the Wnt/β-catenin pathway, we have not explored whether ICA directly binds to Wnt ligands, Frizzled receptors or related proteins. We are interested in this scientific question and will identify the direct upstream target of ICA in our follow-up research. Second, although our existing experimental data have sufficiently clarified the anti-tumor efficacy and underlying molecular mechanism of ICA, the present study lacks *in vivo* animal verification confirming that ICA hinders CRC progression via modulating the Wnt/β-catenin pathway. We will prioritize this research direction in our follow-up experimental design and further investigations.

## Conclusion

ICA effectively reduced tumor number and size, ameliorated intestinal mucosal damage, and increased the expression of genes and proteins related to the mitochondrial-mediated apoptosis through the Wnt/β-catenin signaling pathway in CRC mice. Additionally, *in vitro*, ICA significantly inhibited the proliferation of CRC cells and promoted cellular mitochondrial-mediated apoptosis. The mechanism by which ICA regulates mitochondrial-mediated apoptosis was further proven to be consistent with the *in vivo* results. This study offers novel insights into the molecular mechanisms underlying ICA therapy for CRC, indicating that ICA may be advantageous for the prevention and treatment of CRC.

## Data Availability

The data presented in the study are deposited in the GEO repository, accession number PRJNA1493815.
